# Childhood growth hormone treatment in women with Turner syndrome - benefits and adverse effects

**DOI:** 10.1038/s41598-019-52332-0

**Published:** 2019-11-04

**Authors:** Tomasz Irzyniec, Wacław Jeż, Katarzyna Lepska, Izabela Maciejewska-Paszek, Jakub Frelich

**Affiliations:** 1 Department of Health Promotion and Community Nursing, Faculty of Health Sciences, Medical University of Silesia, Katowice, Poland; 2 Department of Nephrology/ENDO Hospital of the Ministry of the Interior and Administration, Katowice, Poland; 3 Out-patient Clinic for Women with Turner Syndrome, Specialist Hospital No 2, Bytom, Poland; 4 Specialist Dental Clinic S.C, Żory, Poland

**Keywords:** Drug safety, Multihormonal system disorders

## Abstract

Turner syndrome (TS) is characterized by the partial or complete loss of one sex chromosome and results in growth failure, gonadal insufficiency and cardiac anomalies. Treatment with growth hormone (GH) during childhood has indisputable benefits when taking into account the low stature of TS women. Medical records and biochemical findings of 33 TS women treated with GH in childhood (GH+) were compared to those of 124 TS women who did not receive GH (GH−). It seems that the GH-treated group might have had a more severe initial phenotype than the untreated group, as evidenced by higher FSH, more feeding issues in infancy, more lymphedema cases and urinary system malformations. GH+ women were significantly taller and had a better lipid profile and lower prevalence of arterial hypertension than GH− . However, they also had lower thrombocyte counts, a greater prevalence of retrognathism and nail anomalies, especially when the GH treatment was delayed. Long-term GH use was not as effective for growth as GH treatment during the initial period and seemed to have resulted in elevated creatinine levels. GH treatment in childhood has benefits in adulthood; however, adverse effects may occur, especially in individuals with treatment that is delayed or is too long.

## Introduction

Turner syndrome (TS) is characterized by the loss of all or part of one sex chromosome and occurs in 1 in 2500 live-born girls, leading to growth failure, gonadal insufficiency, and cardiac anomalies^1^. The clinical presentation of TS has not been fully characterized^2^. Anatomical and physiological abnormalities of TS can be mild or absent. Clinical features are roughly in parallel with the magnitude of the deficit of X-chromosome material^3^. However, only some of these features are associated with karyotypic differences^4^. In adult women, the diagnosis of TS-related complications is often delayed^5,6^. Women with TS are substantially shorter and have higher body mass indices (BMIs) than their healthy counterparts. They also have a reduced life expectancy, mostly due to cardiovascular diseases^7^. Hormone therapy in TS patients using growth hormone (GH) and sex hormones provides treatment not only for short stature and sex hormone deficiency but also for other effects of TS, including cardiovascular diseases^8^. The patient’s condition at the initial assessment is a result not only of TS alone but also of the treatments received.

GH plays a very important role in maintaining the homogeneity of tissues during normal development or after injury. Its effects on growth during childhood are mediated by insulin-like growth factor 1 (IGF-1)^9^. It is widely acknowledged that in acromegaly, excessive GH causes hypertensive and diabetogenic effects. GH replacement therapy also induces insulin resistance^10^. As a pleiotropic hormone, GH has a number of endo-, auto-, and paracrine effects on practically every tissue. Devesa *et al*. proposed that GH might be a prohormone rather than a hormone since it is proteolytically cleaved in a tissue-specific manner, giving rise to shorter GH forms whose activities are still unknown^9^. Considering the short stature of women with TS, treatment with GH seems to have indisputable benefits even if associated with some metabolic side effects. However, it should be noted that short stature in TS is not related to the deficiency of endogenous GH^11^.

Our previous study revealed a beneficial effect of childhood GH treatment on the lipid profile in adulthood. No negative impact on glucose metabolism was observed^11,12^. Other researchers have also found both direct and long-term positive impacts of GH treatment not only in women with TS but also with regards to cardiovascular diseases^13^.

At present, recombinant human growth hormone (rhGH) is a standard part of childhood treatment for TS. We began our observation in 1995, and our group of TS patients includes women who were not treated with GH in their childhood.

Our study addresses the nongenital phenotypic differences between TS patients that were treated (GH+) and not treated (GH−) with GH in their childhood. Although the benefits of the pediatric application of GH therapy are well established and no mortality has been observed for GH− treated TS children, some long-term adverse effects cannot be excluded^14–16^.

## Results

### TS patients treated and not treated with GH in their childhood

#### karyotypes and HRT

The karyotypes were revealed in the GH+ and GH− groups of women with TS and are shown in Table 1. Isochromosome X or its segments were found in 4 (12.1%) and 23 (18.5%) TS women, respectively (*P* = 0.3861). The proportion of particular karyotypes in the GH+ and GH− groups was comparable, and so was the proportion of women currently receiving HRT, i.e., 60.6% (20 women) in the GH+ group and 54% (67 women) in the GH− group (*P* = 0.4979).

**Table 1:** Karyotypes of 157 women with Turner syndrome treated (GH+) or not treated with growth hormone in childhood (GH−), as revealed in their medical histories. V – absolute values, (%) – percent.

Karyotype	GH+(n = 33)		GH−(n = 124)		GH+ vs GH−
	V	(%)	V	(%)	*P* - value
Simple X monosomy (45,X)	20	60.6	72	58.1	0.7955
Structural abnormalities of the X chromosome (e.g. isochromosome X, partial deletion of an arm of the X chromosome, ring X chromosome or translocation between the X chromosome and the autosomal material)	5	15.1	12	9.7	0.3751
Mosaic karyotypes	8	24.3	40	32.2	0.3812
a. X monosomy and normal female cell line	3	9.1	13	10.5	0.8134
b. X monosomy and normal male cell line	2	6.1	5	4	0.6028
c. X monosomy and a second aneuploid cell line (trisomy)	1	3	1	0.8	0.3144
d. X monosomy and a second cell line with structural chromosome abnormality	2	6.1	21	16.9	0.1187
Sum	33	100	124	100	

#### clinical characteristics

The clinical characteristics of the study subjects are presented in Table 2. Women of the GH+ group were taller (approximately 5 cm), slightly younger (approximately 4.4 years) and had a lower body mass index and better lipid profile (lower total and LDL cholesterol as well as triglyceride levels) than the GH− participants. Additionally, statistically significant differences were noted between these two groups with respect to FSH levels and some blood count parameters (thrombocytes).

**Table 2:** Clinical and laboratory characteristics of 157 women with Turner syndrome (TS) treated (GH+) or not treated with growth hormone in childhood (GH−). Shown are the mean ± SD, ranges are shown in brackets, and normal ranges are shown in square brackets for laboratory investigations.

Parameters [normal ranges]	GH + (n = 33)	GH− (n = 124)	GH + vs GH− *P* - value
Age years	21.8 ± 3.49 (18–31)	26.2 ± 7.9 (18–53)	0.016
Height cm	148.3 ± 6.57 (136–161.5)	143.4 ± 6.15 (123–167.5)	0.0001
Weight kg	47.7 ± 7.84 (37.1–70.8)	50.1 ± 9.7 (34–76.1)	0.2
BMI kg/m^2^	21.7 ± 3.7 (16.8–34.4)	24.4 ± 4.4 (16.8–38.1)	0.0019
Age at final TS diagnosis y.	10.1 ± 5 (1–25)	15.3 ± 8.1 (1–44)	0.0008
FSH IU/L [2.8–11.3]	50.2 ± 26.7 (0.5–132)	34 ± 20.6 (0.5–60)	0.0021
FSH > 40 IU/L %	63.6	40.3	0.017
LH (IU/L) [1.1–77]	27.2 ± 17.7 (1–93)	22.5 ± 16.4 (1–81)	0.2
E_2_ (pmol/L) [66–884]	152.6 ± 161 (45.9–598)	106.6 ± 119 (36.7–838.2)	0.0649
E_2_ < 110 pmol/L %	39.4	28.2	0.2
Cholesterol mmol/L [3.9–5]	4.6 ± 0.88 (3–6.6)	5.1 ± 1.04 (2.8–10.7)	0.0256
HDL mmol/L [ > 0.91]	1.34 ± 0.37 (0.8–2.3)	1.5 ± 0.48 (0.5–3)	0.1
LDL mmol/L [ < 3.88]	3 ± 0.79 (1.5–4.9)	3.3 ± 0.9 (1.4–7.1)	0.049
TG g/L [0.4–1.5]	0.9 ± 0.47 (0.2–2.9)	1.1 ± 0.62 (0.4–4)	0.046
Glucose mmol/L [3.9–5.6]	5.1 ± 0.67 (3.7–6.4)	5.1 ± 0.89 (3.2–10.6)	0.85
Creatinine µmol/L [60–130]	62 ± 10.4 (41.9–81.4)	59.8 ± 14.7 (33.8–161)	0.4
Protein g/L [60–80]	73.9 ± 6.8 (53.5–82.5)	73.6 ± 4.9 (60–86)	0.7
Hematocrit % [40–50]	44 ± 2.6 (39.6–50)	44.2 ± 3.6 (33.9–59)	0.7
Hemoglobin g/dL [12–16]	14.2 ± 0.9 (12.2–16.3)	14.3 ± 0.9 (11.6–17)	0.7
Red blood cells 10^12^/L [4.2–5]	4.66 ± 0.3 (4.2–5.2)	4.72 ± 0.5 (3.3–6.2)	0.4
White blood cells 10^9^/L [4–8]	6.5 ± 1.4 (3.2–9.3)	7.1 ± 2 (3.6–13.9)	0.1085
Thrombocytes 10^9^/L [150–300]	228.6 ± 56.2 (114–329)	259.3 ± 72.5 (113–485)	0.0249

#### medical histories and findings of physical examinations

Differences in the medical histories and findings of physical examinations between the GH+ and GH− women are shown in Tables 3 and 4, respectively. The prevalence of dense eyebrows, long eyelashes, neck anomalies (short, webbed), and blood pressure ≥ 140/90 mmHg was higher in women without GH treatment. A higher proportion of childhood lymphedema and feeding problems during infancy was observed in the GH+ women. However, these features were not GH dependent. A significantly higher prevalence of retrognathism, nail anomalies and/or ingrowing nails as well as an increased prevalence of urinary system anomalies were also noted in GH+ women. The other tables present the parameters characteristic of a GH treatment duration of less than three years (GH < 3) or equal to or longer than 3 years (GH ≥ 3) and those revealed in patients who started GH therapy before the age of 12 years (GH < 12) or at the age of 12 or later (GH ≥ 12).

**Table 3:** Abnormalities in 157 women with Turner syndrome treated (GH+) and not treated with growth hormone in childhood (GH−), as revealed in their medical histories. V – absolute values, (%) – percent.

Parameter	GH + (n = 33)		GH− (n = 124)		GH + vs GH− *P* - value
	V	(%)	V	(%)	
Skin diseases	9	27.3	30	24.2	0.7
Ingrowing nails	12	36.4	23	18.5	0.028
Childhood lymphedema	15	45.5	28	21.7	0.0068
Feeding problems during infancy	23	69.7	61	49.2	0.036
Third tonsil adenotomy	10	30.3	36	29	0.9
Dental braces	6	18.5	23	23.4	0.5
Otitis media	24	72.7	84	67.7	0.6
Surgery for otitis media	3	9.1	15	12.1	0.6
Heart defect(s)	7	21.2	12	9.7	0.072
Urologic surgery	0	0	5	4	0.2
Urinary system malformations	10	30.3	18	14.5	0.035
Conservative kidney management	12	36.4	35	28.2	0.4
History of vision defects	18	54.5	76	61.3	0.5
History of thyroid diseases	5	15.2	23	18.5	0.7
Diabetes mellitus	2	6.1	8	6.5	0.9

**Table 4:** The somatic anomalies and clinical features in 157 women with Turner syndrome treated (GH+) or not treated with growth hormone in childhood (GH−), as revealed by physical examination. V – absolute values, (%) – percent.

Parameter	GH + (n = 33)		GH− (n = 124)		GH + vs GH− *P* - value
	V	(%)	V	(%)	
Pigmented nevi	33	100	122	98.4	0.5
Hypertrichosis on the face	4	12.2	7	5.6	0.2
Low-set and/or deformed ears	29	87.9	103	83.1	0.5
Dental caries and lost teeth	1	3	8	6.5	0.4
High-arched palate	29	87.9	110	88.7	0.9
Malocclusion	17	51.5	57	46	0.6
Retrognathism	26	78.8	71	57.3	0.024
Oral cavity soft tissue abnormalities	2	6.1	3	2.4	0.3
Drooping eyelids	13	39.4	64	51.2	0.2
Epicanthal folds	12	36.4	43	34.7	0.9
Dense eyebrows, long eyelashes	14	42.4	92	74.2	0.0005
Squint	5	15.2	17	13.7	0.8
Daltonism	0	0	2	1.6	0.5
Low hairline on the nuchae	21	63.6	70	56.5	0.5
Neck anomalies (short, webbed)	13	39.4	73	58.9	0.046
Posture defects	16	48.5	53	42.7	0.6
Finger deformity	9	27.3	32	25.8	0.9
Fingernail anomalies	22	66.7	59	47.5	0.0498
Blood pressure ≥ 140/90 mmHg	4	12.2	36	29	0.049

### GH treatment duration

No significant differences were observed between the GH ≥ 3 and GH < 3 subgroups regarding growth, BMI and lipid metabolism parameters (Table 5). The subgroup with shorter exposure (GH < 3) did not exhibit any beneficial effects of the therapy on lipid metabolism, ie., HDL concentrations were comparable to those of the GH− participants. Compared to GH−, TG concentrations were only lower in the GH ≥ 3 subgroup. The platelet and red blood counts were significantly lower in the GH < 3 participants compared to GH ≥ 3; lower number of erythrocytes was associated with an increase in mean corpuscular volume (MCV) and a decrease in mean corpuscular hemoglobin (MCH) as presented in Table 6. The proportion of hypertensive individuals was significantly lower than among the GH− participants. The GH ≥ 3 subgroup had higher creatinine levels than GH−. There was no significant difference regarding the prevalence of clinical TS features between the GH ≥ 3 and GH < 3 subgroups.

**Table 5:** Laboratory parameters, somatic anomalies and clinical features differentiating between 33 women with Turner syndrome treated or not treated with growth hormone in childhood; GH therapy duration <3 years or ≥3 years. V – absolute values and (%) – percentages. ^Superscript *P*-value^ statistically significant compared to TS women not treated with growth hormone in childhood.

Parameters [normal ranges]	GH < 3 (n = 18)	GH ≥ 3 (n = 15)	GH < 3 vs GH ≥ 3 *P* - value
Height (cm)	148.5 ± 6.65^0.0387^	148 ± 6.7^0.0105^	0.8649
BMI (kg/m^2^)	21.8 ± 4^0.0139^	21.6 ± 3.3^0.0064^	0.9136
Time of GH exposition (y)	1.5 ± 0.6	5.27 ± 1.5	0.0000
FSH (IU/L) [2.8–11.3]	49.9 ± 23^0.0076^	50.5 ± 32^0.0595^	0.7255
E_2_ (pmol/L) [66–884]	123 ± 135	188 ± 187	0.7448
Cholesterol (mmol/L) [3.9–5]	4.57 ± 0.93	4.63 ± 0.84	0.8654
HDL (mmol/L) [>1]	1.28 ± 0.25	1.46 ± 0.47	0.1605
LDL (mmol/L) [<3]	3 ± 0.94	3 ± 0.58	0.9962
TG (g/L) [0.4–1.7]	0.96 ± 0.5	0.82 ± 0.3^0.0498^	0.3838
Creatinine (µmol/L [60–130]	59 ± 12	65.5 ± 11^0.0356^	0.0707
Red blood cells (10^12^/L) [4.2–5]	4.57 ± 0.26	4.77 ± 0.28	0.0424
Thrombocytes (10^9^/L) [150–300]	208.5 ± 52^0.0049^	252.8 ± 41	0.0117
	**V (%)**	**V (%)**	
Retrognathism	14 77.8	12 80	0.8776
Fingernail anomalies	13 72.2^0.05^	9 50	0.1905
Ingrowing nails	7 38.9^0.0474^	5 33.3	0.7391
Blood pressure ≥ 140/90 mmHg	1 5.6^0.0345^	3 20	0.2073
Urinary system malformations	5 27.8	5 33.3^0.06^	0.7321
HRT	12 66.7	8 53.3	0.4328

**Table 6:** Red blood cell parameters (mean ± SD) in women with Turner syndrome treated with growth hormone in childhood; GH therapy duration <3 years or ≥3 years. MCV – mean corpuscular volume, MCHC – mean corpuscular hemoglobin concentration, MCH – mean corpuscular hemoglobin. Ranges are shown in brackets.

Parameter	<3 years (n = 18)	≥3 years (n = 15)	*P* - value
Red Blood Cells (T/l)	4.57 ± 0.26 (4.2–4.9)	4.77 ± 0.28 (4.3–5.2)	0.0424
MCV (fL)	95.1 ± 4.62 (86–103)	90.9 ± 3.5 (87.8–99)	0.0422
MCHC (pg/L)	30.3 ± 1.3 (28.5–32.7)	30.7 ± 1.32 (28.2–32.3)	0.4576
MCH (pg/L)	31.9 ± 1.39 (29.7–34.5)	33.8 ± 1.4 (31.3–36.1)	0.0091

### GH treatment initiation

Table 7 shows that, strictly according to expectations, the mean age of GH treatment initiation was lower in the GH < 12 group than in the GH ≥ 12 group. No significant differences were noted regarding the somatometric parameters. Compared to the GH− group, the GH < 12 subgroup had better lipid metabolism parameters (lower levels of total cholesterol and TG), while lower platelet counts were noted in the GH ≥ 12 participants. Only the GH ≥ 12 subgroup had a higher prevalence of retrognathism and ingrowing nails than GH− women.

**Table 7:** Laboratory parameters, somatic anomalies and clinical features differentiating 33 women with Turner syndrome treated with growth hormone in childhood; GH therapy initiation before the age of 12 (GH < 12 years) or later (GH ≥ 12 years). V – absolute values and (%) – percentages. ^Superscript **P**-value^ statistically significant compared to TS women not treated with growth hormone in childhood.

Parameters [normal ranges]	GH < 12 (n = 12)	GH ≥ 12 (n = 21)	GH < 12 vs.GH ≥ 12 *P* - value
Height (cm)	147.1 ± 5.9^0.0387^	149 ± 6.9^0.0009^	0.5474
BMI (kg/m^2^)	21.6 ± 3.7^0.0036^	21.7 ± 3.8^0.0036^	0.9193
Age of start GH treatment	8.25 ± 1.5	14.4 ± 1.9	0.000
FSH (IU/L) [2.8–11.3]	52.4 ± 31^0.0452^	48.9 ± 24^0.0111^	0.7255
E_2_ (pmol/L) [66–884]	152 ± 158	153 ± 175^0.0329^	0.9923
Cholesterol (mmol/L) [3.9–5]	4.43 ± 0.72^0.0234^	4.69 ± 0.97	0.4266
HDL (mmol/L) [>1]	1.3 ± 0.3	1.4 ± 0.4	0.4172
LDL (mmol/L) [<3]	3 ± 0.51	3 ± 0.92	0.9902
TG (g/L) [0.4–1.7]	0.81 ± 0.4^0.059^	0.95 ± 0.5	0.4189
Creatinine (µmol/L) [60–130]	64 ± 11.5	60.3 ± 9.5	0.2329
Red blood cells (10^12^/L) [4.2–5]	4.77 ± 0.24	4.59 ± 0.29	0.0812
Thrombocytes (10^9^/L) [150–300	252.3 ± 34.6	215.1 ± 55.6^0.0087^	0.0453
	**V (%)**	**V (%)**	
Retrognathism	9 75	17 81^0.0397^	0.6849
Dense eyebrows, long eyelashes	5 41.7^0.0174^	9 42.9^0.0039^	0.9465
Fingernail anomalies	8 66.7	14 66.7	1
Ingrowing nails	4 33.3	8 38.1^0.0426^	0.7694
Blood pressure ≥ 140/90 mmHg	1 8.3	3 14.3	0.6114
Urinary system malformations	4 33.3	6 28.6	0.7775
HRT	5 41.7	15 71.4	0.093

## Discussion

The selected medical history, physical examination and biochemical findings of women with TS who did or did not receive GH treatment in childhood were compared for corresponding parameters. Prior to discussing the results obtained in this study, attention should be paid to its limitations. Doubts may be related, for example, to the comparability of analyzed groups. The proportion of women with 45,X monosomy, structural aberrations in the X chromosome and mosaic karyotypes was comparable in both groups. Differences in the deficit of genetic material cannot be ruled out with certainty. For instance, genetically pure 45,X monosomy is considered lethal. Although not yet proven, it is believed that patients with the 45,X blood karyotype have some degree of mosaicism to maintain viability^4^. Despite comparable proportions of women on HRT in both groups, it is possible that the groups were heterogeneous in this respect. We found significant differences in FSH levels in selected groups of TS women. Clinical features were selected based on the literature descriptions of tissue and organ abnormalities seen in women with TS. We aimed to present a wide range of features concerning various systems and, in this context, show differences between the examined groups, realizing that the prevalence of some of these features, e.g., otitis media, feeding problems during infancy, and childhood lymphedema, could not have been related to GH treatment, and, likewise, urinary and heart malformations. The study covered adults with a several-year span between treatment discontinuation and study entry. However, it may have been that the GH− treated group had a more severe phenotype than the untreated group, as evidenced by more feeding issues in infancy and more lymphedema cases. Despite the extensive experience of the research team (the investigations were conducted over a period of 20 years), several of our findings were likely the results of detection bias. Despite all these doubts concerning possible intergroup differences, we decided to proceed with comparison. It is obvious that the obtained results must be interpreted with caution.

In our observations, the benefits of childhood GH therapy manifested themselves as significant increases in body height, lower BMIs, more favorable lipid metabolism parameters and a lower prevalence of hypertension. The potentially negative outcomes of childhood GH treatment include lower thrombocyte counts as well as a significantly higher prevalence of retrognathism and nail anomalies and/or ingrowing nails. The GH+ group also had higher FSH levels, a higher percentage of TS women with FSH values exceeding 40 IU/L and a lower prevalence of dense eyebrows and long lashes. A significantly higher prevalence of urinary system anomalies was also noted.

Longer childhood exposure to GH did not result in greater body height. Despite the lack of effect on growth, longer exposure to GH in childhood had a beneficial long-term effect on lipid metabolism; the red blood cell count was also higher. However, GH ≥ 3 participants exhibited elevated creatinine levels compared to GH− participants. No relationship was found between the prevalence of clinical features and the duration of GH exposure.

The patient’s age at the start of GH therapy did not have any effects on somatometric parameters. GH treatment did not have beneficial effects on lipid metabolism, while therapy initiation after the age of 11 seemed to cause a decrease in platelet counts. Delayed GH treatment was correlated with the prevalence of retrognathism and ingrowing nails.

As mentioned above, though GH has important effects on metabolism, being a pleiotropic hormone, it also has a number of endo-, auto-, and paracrine effects on practically every tissue or organ^9^. The fact that our GH− treated study participants were significantly taller than their nontreated counterparts proves that treatment was effective. The effectiveness of this treatment was within the expected range. According to Gravholt *et al*., 2017 and the International Turner syndrome guidelines, the mean response to GH therapy is 5–8 cm and up to 17 cm with high dose therapy^2^. The equivalent effect on height of a mean of 1.5 years vs. 5.3 years of GH in our two groups may be hard to believe but it may still be the most important point of the paper. BMIs were also significantly lower in the GH+ women. A small, but statistically significant age difference between the GH+ and GH− women might partly account for the differences in clinical and laboratory characteristics.

International guidelines and analyses on GH use in women with Turner Syndrome do not mention retrognathism and skin adnexa abnormalities as a complication of this treatment^2,16^.

Changes in craniofacial morphology are among the most frequently described symptoms of TS^17,18^. Their prevalence in our study is consistent with that reported in the literature, though the proportion of retrognathism was higher in the GH+ women. The effects of hormonal therapy on the development of the craniofacial skeleton differ depending on the age at the start of therapy, dose, etc. Contrary to the effect on long bones, GH treatment started after the age of 7 will not make up for the underdevelopment of craniofacial structures. Therefore, craniofacial changes frequently require orthodontic treatment. The higher proportion of retrognathism in our GH+ participants might be suggestive of a delay in starting the GH treatment^19,20^. Our results showing a relationship between therapy delay and the prevalence of retrognathism seem to confirm this hypothesis. Childhood GH treatment seems to have had opposite effects with respect to the proportion of dense eyebrows and long lashes and the occurrence of nail anomalies and/or ingrowing nails (Tables 3 and 4). Nevertheless, it could be hypothesized that childhood GH treatment can result in troublesome skin adnexal alterations later in life. Skin adnexal abnormalities might also be due to congenital lymphedema. Since the pathogenesis of these complications could be multifactorial, the involvement of GH should not be disregarded. Lichen planus-like reaction during hrGH therapy was described by Soares and Mendonca in 2016. Spontaneous remission occurred after therapy suspension^21^.

Adult changes in erythrocytes and platelet counts might be one of the major clinical concerns when analyzing GH treatment in the childhood of TS patients. Several reports have suggested a role for GH in the regulation of the hematopoietic system. Meazza *et al*. demonstrated that, during the first year of GH treatment, erythropoietin levels decreased significantly in short children without GH deficiency^22^. Kawa *et al*. showed that GH treatment in children with GH deficiency increased the clonogenic growth of erythroid lineage cells and RBC counts and significantly upregulated cell-cycle-propagating genes^23^. This could account for the significantly lower (although still within normal ranges) erythrocyte and thrombocyte counts observed in GH+ women. In another publication, the same team of researchers suggested that recombinant human GH had a direct anti-apoptotic activity in hematopoietic CD34+cells derived from GH− deficient patients^24^. No similar studies have been performed in patients without GH deficiency, e.g., patients with Turner syndrome. While a decrease in platelet count was noted in GH+ compared to GH− patients with TS, lower red blood cell counts with higher MCV and lower MCHC were found in the subgroup with lower GH exposure (GH < 3), which might have some pathogenetic significance.

GH affects renal function and kidney growth both in physiological and pathological conditions^25–28^. Higher creatinine concentrations in long-term users of GH in childhood (GH ≥ 3) than in those not using GH (GH−) might indicate a compromise of excretory kidney function in patients with long-term childhood exposure to GH.

The GH+ women had significantly higher prevalence of urinary malformations compared to the GH− group. How could this be accounted for? Urinary malformation in childhood can prompt genetic testing and early identification of a TS karyotype, which is inherently associated with congenital renal malformations^29,30^. These children had an increased chance for GH treatment. It might also be speculated that a more severe initial TS phenotype prompted GH therapy initiation and more detailed urinary system checks. Hence the GH+ group showed a trend towards higher rates of cardiac and urogenital abnormalities as well as higher proportion of patients who declared lymphedema and feeding problems during infancy.

Apart from its direct or IGF-1-mediated effects on the heart^31–33^, GH also acts at the vascular level^7,8^. A significantly lower proportion of hypertensive women in the GH+ group indicates a beneficial effect of GH on the blood vessels of our GH+ TS patients, which however, does not necessarily translate into better functioning of a damaged heart. Improvements in the lipid profiles of GH+ women favorably influence their cardiovascular system^11^. The almost two-fold decrease in the proportion of heart defects found in our study compared to world literature on the subject^34,35^ might have resulted from the fact that women with severe cardiovascular and hypertensive complications did not accept an invitation to participate in the study.

Finally, it seems important to comment on the significantly higher FSH levels and the percentage of patients with FSH > 40 IU/L in GH+ group as compared to GH−, which was difficult to account for. In our previous study, we reported that a large percentage of women with TS did not use HRT; those who did used this method of treatment insufficiently, i.e., HRT doses were most likely too low^36^. However, some local effects of GH or GH degradation products on the secretion of other anterior pituitary hormones cannot be ruled out.

### Summary

Differences between GH+ and GH− groups with respect to somatic abnormalities, medical history, physical examination findings and laboratory results in women with TS helped to identify features that seem to indicate beneficial long-term effects of childhood GH therapy. Other findings indicate some possible adverse effects of this treatment. We have shown that GH treatment in girls with Turner syndrome improves height, BMI, vascular markers, and lipids. However, these patients also had lower platelet counts as well as a higher prevalence of retrognathism and nail anomalies, especially when the treatment was delayed. Long-term GH use is not as effective for growth as during the initial treatment period. Moreover, it seemed to have resulted in elevated creatinine levels. One major concern is the clinical relevance of the changes in blood count parameters (RBCs, thrombocytes). Another concern is that there seems to be some difference between the two groups, namely, the GH treated group might have had a more severe initial phenotype than the untreated group, as evidenced by higher FSH, more feeding issues in infancy and more lymphedema cases.

## Conclusions

Apart from the beneficial effect of GH on stature, especially at the beginning of treatment of TS women, childhood GH therapy favorably affects the cardiovascular system via improvements in the lipid profile and a decreased prevalence of arterial hypertension. Unlike untreated women, women treated with GH in childhood exhibited lower thrombocyte counts and a greater prevalence of retrognathism, nail anomalies and urinary system malformations. Some effect on the excretory function of the kidneys cannot be excluded. The differences in laboratory findings and the prevalence of some clinical features observed in our GH+ and GH− participants with TS might have resulted from therapy that was too long or delayed. Therefore, further studies are warranted to identify potential drawbacks of pediatric GH treatment.

## Methods

The study population comprised 33 women with TS who were treated with GH (GH+), aged 21.8 ± 3.5 years, and 124 women with no history of GH treatment, aged 26.2 ± 7.8 (GH−). The diagnosis of TS was confirmed by karyotyping using cytogenetic and advanced molecular analysis^5^. Information about the current HRT and previous GH use was obtained from the medical records and each patient’s medical history. The approximate age at the start of GH treatment ranged from 7 to 17 years (mean 12 ± 3.5). The mean duration of GH therapy was 3.5 ± 2.4 years, ranging from 1 to 8 years. The growth-rate-adjusted GH doses were 0.33 to 0.47 mg/kg per week.

Features to be assessed from the patient history and physical examinations were selected based on the literature descriptions of tissue and organ abnormalities seen in women with TS excluding gynecological disorders^3,17^. The investigations were conducted by the same group of investigators over a period of 20 years. The following conditions were evaluated prospectively based on the patient medical history and records: childhood lymphedema, feeding problems during infancy, skin diseases, thyroid diseases, diabetes mellitus, recurrent otitis media and surgery for otitis media, adenoidectomy and vision defects. Cardiovascular and urinary system abnormalities were also considered. On the physical examination, close attention was paid to eyebrow density, eyelash length, drooping eyelids, epicanthal folds, squint, and Daltonism. Low-set and/or deformed ears, high-arched palate, oral cavity soft tissue abnormalities, retrognathism and other types of malocclusion, dental caries and tooth loss were also checked. Neck anomalies (short or webbed) and posture defects were evaluated. We also paid attention to low hairline at the back of the neck, hypertrichosis on the face, pigmented nevi, and the shapes of fingers and fingernails. The prevalence of blood pressure ≥140/90 mmHg was also evaluated. The study participants were examined from March 1995 to September 2015. Gonadotropin (follicle stimulating hormone (FSH), luteinizing hormone (LH)) and 17β-estradiol (E_2_) concentrations were measured in all TS women. FSH and LH levels were determined using a radioimmunoassay, while E_2_ levels were measured using immunoradiometric methods. A complete blood count and blood biochemistry tests were also performed using standard laboratory methods. Body height and weight were measured, and BMIs were calculated for all subjects. To determine the relationship between differences in the mean values of the measurement parameters, the prevalence of somatic anomalies/clinical features and the duration of childhood GH treatment, the GH+ women were divided into those who had received GH ≥ 3 years (n = 15) and those who received GH treatment for less than 3 years (n = 18). Another division was made to evaluate the mean values of the measurement parameters with respect to the age of GH treatment onset. Hence, a subgroup was formed where GH treatment had been initiated before the age of 12 years (n = 12) and another where GH administration had started at the age of 12 or later (n = 21). The results are shown in separate tables.

All procedures performed in studies involving human participants were in accordance with the ethical standards of the institutional research committee and with the 1964 Declaration of Helsinki and its later amendments or comparable ethical standards. All participants gave their consent to study procedures, all of which were approved by the local Bioethics Committee at the Medical University of Silesia.

Statistical analysis was carried out using Statistica 12 software (StatSoft Inc., Palo Alto, CA, USA), and the results are presented as the mean ± standard deviation (SD). We used an unpaired t-test to compare the results. The results of hormonal tests were compared using the Mann-Whitney U test. The bilateral test of differences between two structural indicators was applied to evaluate the statistical characteristics of the frequency of occurrences of particular values. The level of statistical significance was set at *P* < 0.05.

## Data Availability

The datasets generated during and/or analysed during the current study are available from the corresponding author on reasonable request.
